# The Impact of Changing Medicaid Enrollments on New Mexico's Immunization Program

**DOI:** 10.1371/journal.pone.0003953

**Published:** 2008-12-24

**Authors:** Michael A. Schillaci, Howard Waitzkin, Tom Sharmen, Sandra J. Romain

**Affiliations:** 1 Department of Social Sciences, University of Toronto Scarborough, Toronto, Ontario, Canada; 2 Departments of Sociology, and Family & Community Medicine, University of New Mexico, Albuquerque, New Mexico, United States of America; 3 Office of Community Assessment, Public Health Division, New Mexico Department of Health, North Valley Public Health Office, Albuquerque, New Mexico, United States of America; 4 Department of Social Sciences, University of Toronto Scarborough, Toronto, Ontario, Canada; National Institute for Public Health and the Environment, Netherlands

## Abstract

**Background:**

Immunizations are an important component to pediatric primary care. New Mexico is a relatively poor and rural state which has sometimes struggled to achieve and maintain its childhood immunization rates. We evaluated New Mexico's immunization rates between 1996 and 2006. Specifically, we examined the increase in immunization rates between 2002 and 2004, and how this increase may have been associated with Medicaid enrollment levels, as opposed to changes in government policies concerning immunization practices.

**Methods and Findings:**

This study examines trends in childhood immunization coverage rates relative to Medicaid enrollment among those receiving Temporary Assistance for Needy Families (TANF) in New Mexico. Information on health policy changes and immunization coverage was obtained from state governmental sources and the National Immunization Survey. We found statistically significant correlations varying from 0.86 to 0.93 between immunization rates and Medicaid enrollment.

**Conclusions:**

New Mexico's improvement and subsequent deterioration in immunization rates corresponded with changing Medicaid coverage, rather than the state's efforts to change immunization practices. Maintaining high Medicaid enrollment levels may be important for achieving high childhood immunization levels.

## Introduction

New Mexico is a largely rural state with a comparatively high proportion of its population living in poverty (18.5%)[1] and without health insurance (20.3%)[2]. Like other rural states, New Mexico has faced problems in the past with timeliness of childhood immunizations. Previously, we documented a precipitous decline in New Mexico's immunization rates between 1996 and 2001 [3]. This decline resulted in New Mexico dropping from 30^th^ among states in immunization coverage during 1996, to 51^st^ during 2001. Only Louisiana exhibited a greater decline during this period, slipping from 10^th^ among states in 1996 to 50^th^ in 2001.

After 2001 New Mexico experienced a turn-around in immunization rates, increasing from approximately 63% to 83.5% in 2004. As a result, New Mexico's ranking changed from last or near last in the nation to 15^th^. Consequently, New Mexico received the National Immunization Program award from the Centers for Disease Control and Prevention (CDC) as the state that showed the greatest improvement between 2000 and 2004. Since 2004, however, immunization rates in New Mexico declined to 76.2% in 2006 and ranked 46^th^ in the nation [4].

### Changes in the state's immunization program

Since 2001, 4 policy changes focused on New Mexico's immunization program. These changes followed the establishment of immunizations as a public health priority in 2003 [5]. The various initiatives associated with the new public health priority aimed to achieve the state's goal of 90% coverage by 2010. These policy changes included: 1) development of the New Mexico Immunization Coalition; 2) an accelerated “Done-by-One” schedule of recommended immunizations to be accomplished during the first year of life; 3) the Shot Team Nurse Initiative; and 4) the “Shot for Tots to Teens” outreach program. These initiatives, which represented major state-level health policy changes in response to very low coverage levels between 1996 and 2001, became essential components in New Mexico's revitalized immunization program.

#### New Mexico Immunization Coalition (NMIC)

NMIC emerged in the spring of 2003 as a partnership between the DOH and the University of New Mexico's Health Sciences Center. The overall objective was to achieve on-time, age-appropriate immunizations for 90% of New Mexico's children by 2010. NMIC's priorities included: a) support for the implementation of a Statewide Immunization Information System (NMSIIS), b) promotion of public and provider education, c) advocacy and consensus building for sound and dependable immunization policies, d) facilitation of local immunization coalitions and special events including activities during the annual National Infant Immunization Week, and e) an annual provider awards dinner which recognizes providers with at least 90% of their patients up to date on immunizations. NMIC also helped coordinate the annual statewide “Shots for Tots to Teens” day.

#### Accelerated Immunization Schedule

The Done-by-One program, implemented during the spring of 2003, used an accelerated immunization schedule based on the minimal allowable interval between immunizations in a series for any given vaccine. Not including the initial hepatitis B vaccine given at birth, this accelerated program meant that a child received all immunizations — a total of more than 15 vaccines for the currently recommended vaccination series — during the final four recommended well-child visits up to one year of age (i.e., 2, 4, 6 and 12 months). Strict adherence to the accelerated Done-by-One schedule was essential for the diphtheria and tetanus toxoids and acellular pertussis vaccine series, which required 4 immunizations with a minimum 6-month interval.

#### Nurse Intervention Program

The Office of the Governor funded the Shot Team Nurse Initiative through an annual grant. This intervention program, created during the spring of 2004, provided 5 to 6 nurses who reviewed patient records at providers' offices. Children who were not up to date on their immunizations were contacted and asked to come for their missed vaccines. The Shot Team nurses also trained the nursing personnel and other staff members at the provider's offices in “best practices” to increase effectiveness. Preliminary data presented by the DOH indicated that clinical sites receiving a Shot Team intervention have experienced a greater average improvement than those sites without an intervention (23% versus 12%)[5].

#### Outreach and Promotion

The “Shot for Tots to Teens”, created in 2003, offered a 1-day event when providers opened their doors on a Saturday for parents to get their children up to date on immunizations. No appointment was needed, and there was no cost to the parents. Local media organizations, some of which partnered with the NMIC, promoted the event. An annual grant from the Office of the Governor funded the program.

### Changes in Medicaid policies

Through enactment of the Personal Responsibility and Work Opportunity Reconciliation Act in August 1996, the automatic link between welfare and Medicaid eligibility was severed. As part of the new welfare reform law, the Aid to Families with Dependent Children (AFDC) and the Job Opportunities and Basic Skills Training (JOBS) programs — commonly known as “welfare” — were replaced by the Temporary Assistance for Needy Families (TANF) program. TANF provided assistance and work opportunities to needy families by granting states the federal funds to develop and implement their own welfare programs [6].

**Figure 1:**
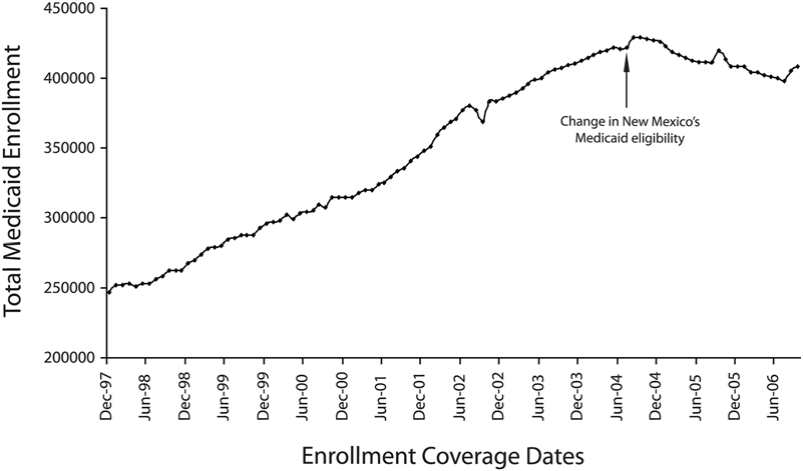
Total Medicaid enrollment levels between 1997 and 2006.

In July 2004, New Mexico implemented 2 Medicaid enrollment changes that may have contributed to a decline in Medicaid enrollments (Figure 1), including those also receiving TANF benefits. First, the state initiated automatic closure of cases that did not complete the eligibility certification process. Second, the state moved from recertification of eligibility every 12 months to every 6 months. After these changes, overall Medicaid enrollment in New Mexico decreased by approximately 4.7%, by far the largest drop during that time period in the nation [7]. In contrast, during this same time period, the average state enrollment nationally increased by 3.2%. A 6.6% decline in enrollment for Medicaid's family and children's categories accompanied New Mexico's overall decrease in Medicaid enrollment [7]. The decline in enrollment proved particularly salient in New Mexico, where children comprised 70.9% of the total Medicaid enrollment — the highest in the nation [7]. This proportion of childhood enrollment was quite high relative to the national average (53.6%).

We investigated the relationship between childhood immunization levels and Medicaid enrollments among those also receiving TANF. Although previous work has assessed the impact of targeted interventions to improve immunization practices in local or regional Medicaid programs [8]–[14], assessment of states' immunization coverage levels linked to specific state-level policy changes has not been as thorough.

## Methods

Data on immunization rates (Table 1) were obtained from the CDC's National Immunization Survey (NIS) [4]. The CDC does not collect data on insurance coverage, nor does any state agency. Specific information on immunization rates by Medicaid enrollment status is, therefore, not available. Data on Medicaid enrollment came from the January 2007 issue of the State of New Mexico's Human Services Department Monthly Statistical Report and the 2005 report from the Kaiser Commission on Medicaid and the Uninsured [7]. We focused on Medicaid enrollment associated with the TANF welfare program in our assessment, rather than overall enrollment. Assessing immunization coverage relative to the number of Medicaid enrollees receiving TANF allowed us to examine the relationship between changes in TANF welfare policy at the state level and the delivery of immunizations, an important component of childhood preventive care.

**Table 1:** National Immunization Survey (NIS), sample sizes (N), estimated number of children 9–35 months of age, and annual estimates of coverage and national ranking for the 4∶3∶1∶3∶3 and 4∶3∶1∶3 series in New Mexico.

Year	N	Children	4∶3∶1∶3∶3	Rank	4∶3∶1∶3	Rank
1996	270	40004	66.2±6.4	30.5	77.6±5.7	26
1997	293	39403	66.1±6.3	38	72.7±6.1	41
1998	254	39573	66.0±6.6	47	71.1±6.4	51
1999	298	39335	66.6±6.4	46	73.0±6.1	46
2000	309	39506	64.5±6.1	50	68.2±5.9	51
2001	338	39615	63.2±5.5	51	71.0±5.1	50
2002	252	38824	64.6±6.7	50	67.4±6.6	49
2003	253	38165	75.2±6.8	45	77.0±6.6	46
2004	325	38645	83.5±5.3	15	84.8±5.2	20
2005	203	39094	78.4±6.7	36.5	79.6±6.5	38
2006	N/A	N/A	76.2±5.2	45	76.8±5.2	46

**SOURCE:** National Immunization Survey (see text).

**NOTES:** ±95% Confidence intervals of immunization rates for the 4∶3∶1∶3∶3 and 4∶3∶1∶3 series are shown. N/A indicates not yet available.

The CDC's National Immunization Survey collects data on immunization rates using a random-digit dialing telephone survey of households with children 19–35 months of age followed by a medical provider's record check to confirm a child's up-to-date status. The NIS adjusts for potential survey bias though statistical weighting [15]. Specifically, the NIS employs weighting procedures that adjust for households without telephones, as well as an adjustment for survey bias associated with nonresponse [15].

Annual NIS immunization rate estimates assess the proportion of children between 19–35 months of age who are up-to-date in their immunizations for a given reporting year. That methodology means that not all children 19–35 months of age who were included in the NIS report for any given year were actually immunized that year [3]. For example, children included in the NIS report for 2004 were born between February 2001 and May 2003. Although the American Academy of Pediatrics recommends that childhood immunizations be completed by 18 months of age, parents typically bring their child in to see a primary care provider for the child's 2 year well-child visit. This means that most children included in the NIS report for 2004 received their last childhood immunizations between February 2003 and May 2005. Because the NIS does not include children immunized after December of a given reporting year, the up-to-date immunization coverage reported for 2004 reflected immunizations given over the 23 month period between February 2003 and December 2004 [3].

It is impossible to determine precisely the time lag between when most of the children were immunized and the end of the reporting year [3]. For this study, we estimated this time lag as approximately 12 months. A 12-month time lag roughly corresponds to the temporal mid-point between the beginning and end dates of the reporting period. This estimate of time lag assumes that immunizations occur at approximately similar frequency throughout the reporting period. The time-lagged estimates of immunization rates therefore should be contemporaneous with the estimates of Medicaid enrollment, allowing us to assess potential causal relationships.

We examined temporal trends in immunization rates by using published results from statistical comparisons provided by the CDC and bivariate plots describing trends in rate estimates. The CDC statistical comparisons identify significant differences by using 95% confidence intervals which account for the complexity and potential biases of their survey data [4].

We chose the 4∶3∶1∶3 and 4∶3∶1∶3∶3 series for analysis. The 4∶3∶1∶3 series includes 4 doses of the diphtheria and tetanus toxoids and acellular pertussis vaccines (DTaP), 3 doses of inactivated polio vaccine (IPV); 1 dose of measles, mumps and rubella vaccine (MMR), and 3 doses of *Haemophilus influenzae* type b conjugate vaccine (Hib). The 4∶3∶1∶3∶3 is the same as the 4∶3∶1∶3 series but includes 3 doses of the hepatitis B vaccine (HepB). To assess the relationship between TANF Medicaid enrollment levels and immunization coverage levels, we compared temporal patterns in immunization coverage and Medicaid enrollment using nonparametric Spearman correlation analyses.

## Results

**Figure 2:**
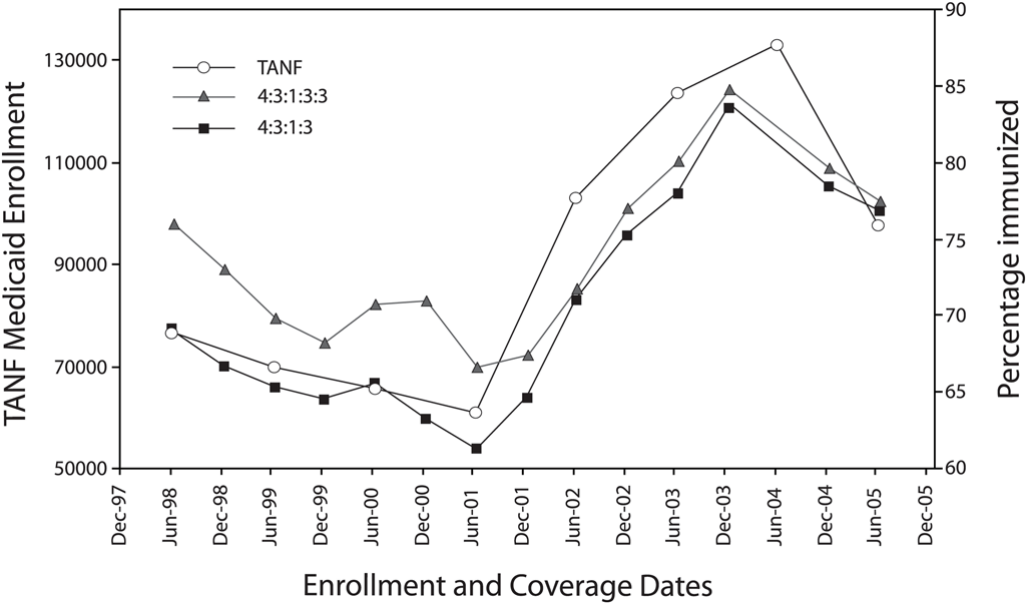
Temporal trends in immunization coverage levels and TANF Medicaid enrollment in New Mexico between June 1998 and June 2005. The NIS immunization coverage estimates are lagged by 12 months. It is important to note that NIS estimates for June 2005 reflecting immunizations in 2004 are not available.

An examination of NIS immunization rate estimates (Table 1) shows a dramatic increase in New Mexico's rates for both immunization series following a fluctuating decline between 1996 and 2002 (Figure 2). The CDC's statistical comparisons of annual rate estimates provides more specifics about the gains in coverage levels that occurred between 2002–2003 and 2003–2004 (Table 2). An approximately 5% decrease in coverage levels for these series occurred between 2004 and 2005. None of the changes in immunization rates were significant statistically.

**Table 2:** Statistical comparisons of observed differences (%) in annual coverage levels by vaccine series.

Series	2001–2002	2002–2003	2003–2004	2004–2005	2005–2006
4∶3∶1∶3	−3.6±8.4	8.8±9.9	7.8±8.4	−5.2±8.4	−2.3±8.4
4∶3∶1∶3∶3	1.4±8.7	10.0±10.0	8.3±8.6	−5.0±8.5	−2.8±8.3

**SOURCE:** National Immunization Survey (see text).

**NOTE:** ±95% Confidence intervals of immunization rates for the 4∶3∶1∶3∶3 and 4∶3∶1∶3 series are shown.

Visual comparison of temporal trends in immunization rates and TANF Medicaid enrollment levels revealed very similar patterns (Figure 2). As TANF Medicaid enrollment levels decreased from June 1998 to June 2001, so did immunization rates, albeit with some minor fluctuation. Similarly, as enrollment levels increased between June 2001 and December 2003, immunization rates improved.

The correlation between TANF Medicaid enrollment levels and immunization rates was highly significant and varied from r_s_ = 0.86 (p = 0.014) for the 4∶3∶1∶3 immunization series, to r_s_ = 0.93 (p = 0.003) for the 4∶3∶1∶3∶3 series.

## Discussion

Our assessment of New Mexico's immunization program suggests that Medicaid enrollment by families under the TANF program may have driven much of the change in childhood immunization rates. Policy changes in 2004 included automatic closure of Medicaid cases not completing the eligibility certification process and required recertification every 6 months. These system-level policy changes affected Medicaid enrollment levels adversely.

Several considerations lead us to believe that changes in TANF Medicaid enrollment played a causal role in changing immunization rates, even though analyses of statistical correlation cannot conclusively demonstrate causation: a) The results identify a compelling temporal correspondence between changes in TANF Medicaid enrollment and changes in childhood immunization levels. b) The causal direction could not plausibly have operated in the opposite direction, that is, by changes in immunization rates leading to changes in Medicaid enrollment. c) We know of no intervening or confounding variables that could account for the very high correlation between changing Medicaid enrollment and changing immunization rates.

Although the various initiatives associated with New Mexico's revitalized immunization program, such as the accelerated “Done-by-one” immunization schedule, represent important changes that aimed to achieve the state's goal of 90% coverage by 2010, these initiatives were implemented too late to be responsible for the dramatic increase in immunization coverage rates between 2001 and 2004. For example, the implementation of both the NMIC and the accelerated “Done-by-one” immunization schedule occured in the spring of 2003, too late to affect substantially the improved immunization rates reported for earlier years. Similarly, the Shot team nurse intervention program was implemented in 2004, and would not have affected rates until the following year.

We suggest, therefore, that expanded Medicaid enrollment levels for needy families played an important role for the increase in New Mexico's immunization coverage levels between 2002 and 2004. Similarly, recent dramatic decreases in TANF Medicaid enrollments contributed to the decrease in immunization rates since 2004.

Because childhood immunizations for the most part are tied to well-child visits, a change in immunization coverage levels may serve as a sentinel for the level of early childhood preventive care. Since the implementation of Medicaid managed care in New Mexico in 1997, primary care practitioners (PCPs) have provided the vast majority of immunizations during the 5 recommended well-child visits before 24 months of age, rather than public health clinics, as was the common practice prior to 1997. For example, in 1997, 39.7% of the nearly 24,000 children in Bernalillo County (New Mexico's largest county) were vaccinated at 1 of 5 public health offices operated by the state's Department of Health (DOH); by 2000 this percentage had dropped to 4.9%. Currently, approximately 2.5% of Bernalillo County's children less than 3 years of age are immunized at public health offices (unpublished data, New Mexico DOH). Consequently, PCPs employed by the managed care organizations that provide preventive care for Medicaid children play an important role in maintaining or improving immunization rates for Medicaid children. Because of New Mexico's high proportion of children enrolled in Medicaid, policy changes that reduce Medicaid enrollment also reduce the likelihood that PCPs will provide adequate preventive care, including immunizations.

Based on our analysis, we believe that increasing the proportion of Medicaid-eligible children who are enrolled in Medicaid and, importantly, who are assigned a PCP, likely affects overall immunization rates, and other aspects of preventive care [16], in a positive way. Our previous study examining the effects of Medicaid managed care on immunization rates did not recognize this relationship [3]. The previous study identified 3 probable causes for declining immunization rates between 1997 and 2002: 1) reduced funding for immunizations at public health offices, 2) informal referrals by Medicaid providers to community health centers and public health offices, and 3) increased workloads at community health centers. That study also suggested that unanticipated and adverse consequences can result from health policy interventions in a complex health system. Our current findings suggest that declining enrollment in the TANF component of Medicaid also may have led to an unanticipated consequence of reduced immunizations.

Several researchers have studied the impacts of decreased TANF enrollment in other states [17]–[19]. Although people who leave TANF usually remain eligible for Medicaid, confusion regarding eligibility and time limits for cash benefits led to a high proportion of uninsurance [20]. In Oregon, for example, 40% of TANF recipients who were disenrolled, including approximately 15–30% of their children, became uninsured after a 1-year transitional Medicaid program ended [21]. Restricting TANF enrollment, therefore, likely represents a barrier to childhood preventive care because children and their families lose their PCPs and medical homes for primary care; as a result, up-to-date status for childhood immunizations declines [22].

### Limitations of the study

The results of our analysis are subject to several limitations. Because individual-level or aggregated data on immunization coverage by insurance status are not available, we were not able to assess directly the affects of Medicaid enrollment on immunization coverage. In addition, research has indicated that the correlation coefficient based on aggregated data can sometimes produce a biased estimate of individual level correlation [23]. It is important to consider, however, that independent of purely quantitative measures, the visible correspondence between immunization rates and Medicaid enrollments is compelling. It seems unlikely that this correspondence is coincidental.

As reviewed by Burns et al.,[24] there are a number of important potential barriers faced by those needing immunizations in addition to insurance status. These barriers include confusion about vaccination schedules, fears about vaccine safety, transportation problems, and inconvenience of the immunization process (e.g., inconvenient clinic hours and long wait times)[24]. In their review, Burns et al.[24] cite an example from Pennsylvania, where after an outbreak of Hib disease, the most commonly cited reason by parents for not having their children immunized was that immunizations were simply not a priority compared to the other activities of life [24], [25]. In that same study, 73% of the parents also indicated they would immunize their children if vaccinations were offered locally [25]. Although our study did not consider these other barriers, it is important to consider that these factors would likely not generate the temporal pattern in immunization rates that we observed. For example, it seems unlikely that there were temporal trends in transportation problems or parental concerns regarding vaccine safety similar to those observed for immunization coverage and TANF Medicaid enrollment between 1998 and 2005.

In addition to the barriers listed above, shortages in vaccine supply can also affect immunization rates [24]. National vaccine shortages for at least one vaccine (pneumococcal conjugate vaccine [PCV]) in 2004 [26], [27] conceivably may have corresponded with the drop in immunization coverage rates in New Mexico during 2004. However, PCV was not among the vaccines comprising the 4∶3∶1∶3 or 4∶3∶1∶3∶3 series examined by our study.

Future research on Medicaid and immunization coverage in poor and rural states such as New Mexico would benefit from the collection of additional survey data at the individual level. Survey instruments should include questions to parents regarding insurance status and other socio-economic information, as well as perceived barriers to immunizing their children such as problems of Medicaid eligibility.

### Conclusions

Subject to certain limitations, our study suggests that deteriorating childhood immunization coverage accompanied state-level changes in welfare policy in New Mexico. As in our previous study [3], [28], the current findings illustrate how unanticipated consequences can follow policy changes in a complex health care system. The prior improvement in immunization coverage in New Mexico, attributed by CDC and others to initiatives by state government to improve immunization practices, probably reflected expansion in Medicaid enrollment. Our research underscores the importance of expanding and maintaining Medicaid enrollment as a key component of efforts to improve immunization coverage as an indicator of public health standards.
